# Complete genome sequence of *Enterobacter ludwigii* strain GW isolated from barley seedling roots

**DOI:** 10.1128/mra.01055-25

**Published:** 2026-06-22

**Authors:** Chandana Pandey, Ajay Madhusudan Sorty, Wenjing Tian, Thomas Roitsch, Peter Stougaard, Athanasios Zervas

**Affiliations:** 1 Department of Plant and Environmental Sciences, University of Copenhagen 4321, Taastrup, Denmark; 2 Department of Environmental Science, Aarhus University 307855, Roskilde, Denmark; University of Maryland School of Medicine, Baltimore, Maryland, USA

**Keywords:** *Enterobacter ludwigii*, plant growth promoting rhizobacteria (PGPR), root growth and architecture

## Abstract

Strain GW, isolated from barley seedling roots, significantly reduced the primary root length formation of barley, *Hordeum vulgare,* and strongly affected root growth and architecture. Oxford Nanopore sequencing and subsequent phylogenetic analyses showed that strain GW was a subspecies of *Enterobacter ludwigii*.

## ANNOUNCEMENT

*Enterobacter ludwigii*, a keystone species of most plant microbiomes, is a multi-functional root endosymbiont combining nitrogen fixation, phosphate solubilization, hormone production, and stress protection (1–3). We report the complete sequence of strain GW, which was isolated from barley seedling roots and strongly inhibits root length, increases root branching, and induces root hairs. This differential impact on the root architecture of barley and wheat genotypes will facilitate mechanistic interaction studies.

**Fig 1:**
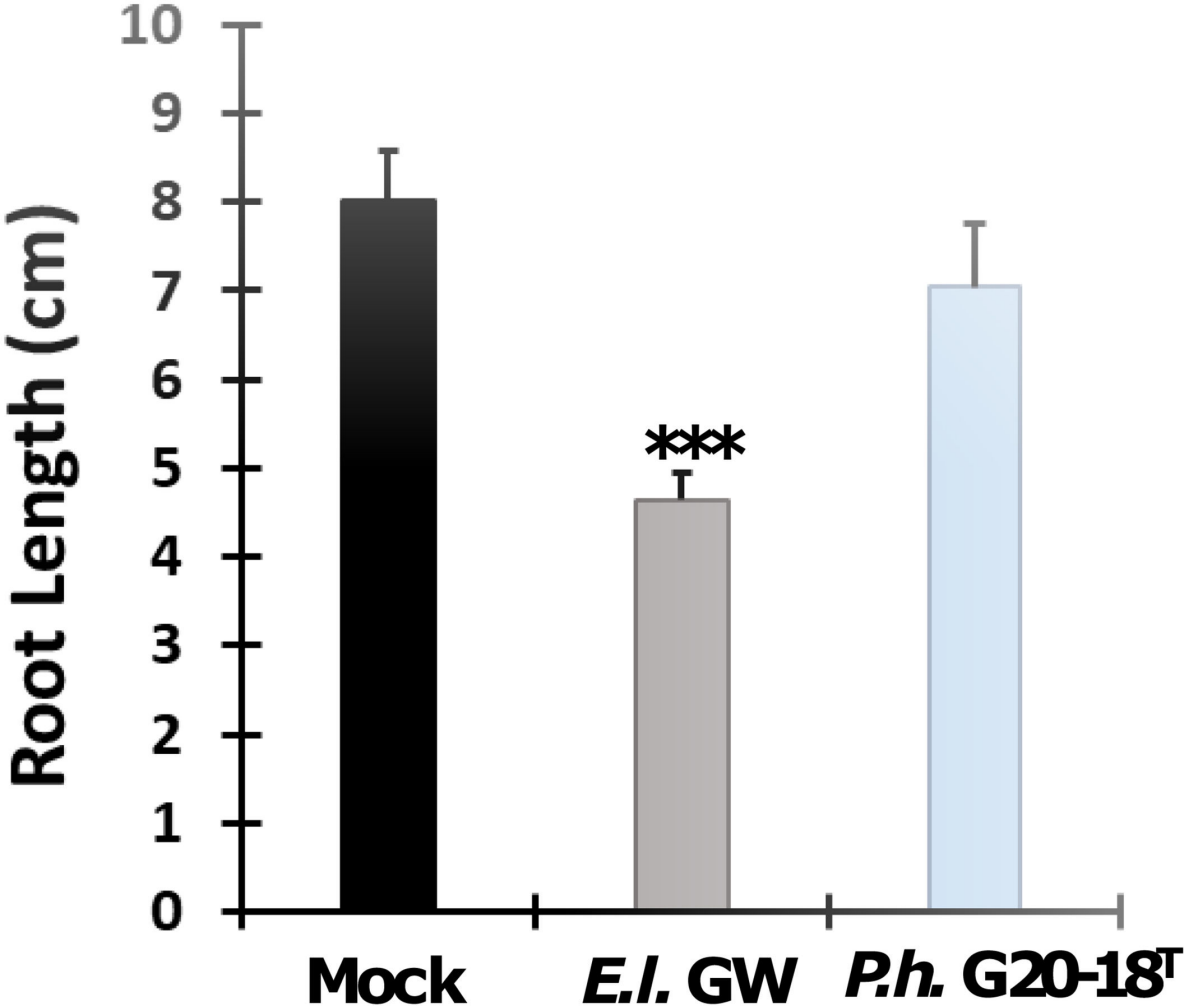
Effect of *E. ludwigii* GW treatment on root length in seedlings. Barley seedlings were treated with either mock solution (Mock), *E. ludwigii* GW (*E.l*. GW), or *P. hormonii* G20-18^T^ for 30 min and then transferred to pre-sterilized germination pouches (16.5 × 17.8 cm) from Mega International (Roseville, MN, USA) with five seeds per pouch and four biological replications. The pouches allow the roots to grow gravitropically along a filter paper matrix (4). The pouch reservoir was filled with 35 mL of the respective solution. Seeds were germinated in the dark for 72 h and then maintained under a 16 h light/8 h dark cycle at 22°C for up to 6 days. Root length was measured 6 days after treatment. Data are presented from *n* = 4 biological replicates. Statistical significance was determined using Welch’s unpaired *t*-test; *** indicates *P* ≤ 0.001 compared with mock.

Strain GW was isolated from seedling roots of cultivar Guld in 2020 from a germination pouch assay (4) at the Taastrup campus (55.67088716°N, 12.30930799°E). Surface-sterilized seeds were germinated in the presence *of P. hormonii* G20-18^T^ (5). Although the growth of most roots was not affected, some were inhibited. Isolation of bacteria from inhibited roots showed, in addition to G20-18^T^, a second type of colony. Purified bacteria representing this second colony type were shown to inhibit root growth (Fig. 1). Phylogenetic analysis by 16S rRNA gene amplification, Sanger sequencing (Eurofins Genomics), and BLASTn comparison showed >99% identity to *Enterobacter ludwigii* EN-119^T^. The isolation from sterilized seeds supports the expected endophytic lifestyle.

Single colonies were grown in LB broth at 28°C for DNA extraction using the MasterPure Complete DNA & RNA Purification kit (Lucigen, Middleton, WI, USA). Oxford Nanopore Technologies libraries were prepared using the Rapid Barcoding Kit 24 V14 (SQK-RBK114.24), without size selection, and loaded on a MinION using an R10.4.1 flow cell. All programs were run with default settings unless otherwise stated. The run was controlled by MinKnow 23.04.5, and basecalling was done by Dorado v.0.5.4, using the super high-accuracy model. In total, 21,360 raw Oxford Nanopore Technologies (ONT) reads were generated. Mean and median read lengths of the ONT reads were 7,213 bp and 3,341 bp, respectively, as calculated by NanoStat v.1.6.0 (6). Low-quality reads, barcodes, and adapters were trimmed from the long reads using Dorado, and *de novo* whole genome assembly was performed using Flye v.2.9-b1768 (7) with the –nano-hq flag and 10 polishing iterations. The assembly graph was visualized using Bandage v.0.8.1 (8), while the quality of the genome was verified using BUSCO v.5.4.0 (9) and its generalized bacteria odb10 (2020-03-06) database. The assembly graph generated by Flye suggests that the sequence was complete and circular. The complete genome was annotated using the NCBI prokaryotic genome annotation pipeline (10) (done by NCBI during submission of the genome). It consists of a chromosome (4,905,758 bp) with a G + C content of 54.5% and does not contain plasmids. In total, 5,215 genes were predicted on the genome, including 85 tRNA, 24 rRNA genes in eight operons, and 5,106 protein-coding sequences. The annotated 16S rRNA genes were used for BLAST searches (blastn) against NCBI’s 16S database, implemented in Geneious Prime 2025.1.3 (Biomatters). The closest related sequences belonged to the genus *Enterobacter*. Average nucleotide identity (OrthoANIu) (https://www.ezbiocloud.net/tools/ani) and *in silico* DNA-DNA hybridization (DDH; GGDC) (https://ggdc.dsmz.de/ggdc.php) analyses showed 98.87% and 91.30% identity, respectively, to the type strain *Enterobacter ludwigii* EN-119^T^, indicating that strain GW is a subspecies of *E. ludwigii* EN-119^T^.

## Data Availability

Raw Oxford Nanopore Technologies long reads were deposited in NCBI at the SRA and are available under the accession number PRJNA1309964. The complete genome sequence of *E. ludwigii* GW is available at NCBI under accession number CP188220.
